# Shorter recovery times and better cognitive function—A comparative pilot study of magnetic seizure therapy and electroconvulsive therapy in patients with depressive episodes

**DOI:** 10.1002/brb3.1900

**Published:** 2020-10-17

**Authors:** Junyan Zhang, Yanping Ren, Wei Jiang, Jiong Luo, Fang Yan, Yilang Tang, Xin Ma

**Affiliations:** ^1^ The National Clinical Research Center for Mental Disorders & Beijing Key Laboratory of Mental Disorders Beijing Anding Hospital Capital Medical University Beijing China; ^2^ Advanced Innovation Center for Human Brain Protection Capital Medical University Beijing China; ^3^ Department of Psychiatry and Behavioral Sciences Emory University School of Medicine Atlanta GA USA; ^4^ Mental Health Service Line Atlanta VA Medical Center Decatur GA USA

**Keywords:** cognitive function, depressive episode, electroconvulsive therapy, magnetic seizure therapy, recovery time of orientation

## Abstract

**Introduction:**

Magnetic seizure therapy (MST) is a new convulsive therapy that is as effective as traditional electroconvulsive therapy (ECT) in treating depression but with fewer cognitive side effects. The aim of this study was to compare the efficacy and cognitive effects between MST (100 Hz applied over the vertex) and bifrontal ECT for treating patients with depressive episodes.

**Methods:**

Forty‐five patients with depressive episodes were enrolled, with 18 receiving MST and 27 receiving ECT. MST was administered over the vertex with 100 Hz frequency. Treatment consisted of six sessions. The 17‐item Hamilton Rating Scale for Depression (HAMD‐17) was used to assess the severity of depression. The Repeatable Battery for the Assessment of Neuropsychological Status (RBANS) was used to assess cognition. Assessments were performed at baseline and after the third and sixth treatment sessions.

**Results:**

Both MST and ECT improved the patients’ depressive symptoms significantly, yet no significant difference was found between the two groups (*p* > .05). The response rates and remission rates of MST and ECT were 72.2% versus 81.5% and 61.1% versus 63.0%, respectively. The MST group showed significant improvements in immediate memory (*p* < .001), delayed memory (*p* = .002), and attention (*p* < .001) than ECT. The recovery times for consciousness (*p* < .001), spontaneous breathing (*p* < .001), and orientation (*p* < .001) were shorter in MST group than ECT group. RBANS improvements were negatively correlated with the recovery time for orientation (*r* = .561, *p* < .001).

**Conclusion:**

Magnetic seizure therapy showed similar efficacy to bifrontal ECT for treating depressive episodes. While MST may be an effective alternative to ECT, larger randomized trials are needed.

## Graphical abstract

The efficacy and cognitive effects between MST and bifrontal ECT for treating patients with depressive episodes were compared. Both MST and ECT improved the patients’ depressive symptoms significantly. MST showed similar efficacy to bifrontal ECT for treating depressive episodes and may be an alternative to ECT.

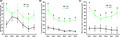

## 1 INTRODUCTION

Depression is a common mental disorder, affecting approximately 4.4% of the world's population (Smith, 2014). In the next 20 years, depression will likely become the leading cause of disability, resulting in higher suicide rates and substantial social and economic burdens globally (Blackburn, 2019). While antidepressants are the most common treatment for depression, only 56% of patients respond positively to these drugs and 44% of patients have treatment‐resistant depression (Bergfeld et al., 2018). In addition, antidepressants do not provide rapid benefits, with most patients requiring 4–8 weeks of continuous therapy to experience benefits (Huda et al., 2018). Electroconvulsive therapy (ECT) is the most effective and rapid treatment for depressive episodes (Hermida et al., 2018), with remission rates of 50%–75% in patients with severe depression. However, the use of ECT is limited due to its adverse effects, including memory loss and cognitive impairments (Bodnar et al., 2016; Napierała et al., 2019).

As an alternative to ECT, magnetic seizure therapy (MST) is a new type of convulsive therapy (Eric et al., 2015). Specifically, MST is a neuromodulation technique that applies high‐frequency repetitive transcranial magnetic stimulation to induce therapeutic seizures (Luber et al., 2013). MST provides directed and focal stimulation, while avoiding the direct stimulation of the deep medial temporal lobe, a region of the brain associated with cognitive impairments in ECT ("Magnetic seizure therapy reduces suicidal ideation and produces neuroplasticity in treatment‐resistant depression" Sun et al.,^2018^). Hence, MST is a promising alternative to ECT, and several studies have verified its efficacy for depression and other mental disorders (Daskalakis etal., 2020; Fitzgerald et al., 2018; Jiang et al., 2018; Tang et al., 2018). However, additional comparative studies are needed to assess the efficacy, systemic, and cognitive adverse effect profiles of MST and ECT.

Based on these previous findings, we conducted this rater‐blinded, controlled study to compare the efficacy and cognitive effects of MST and ECT in Chinese patients with depression. In this study, we aimed to compare the clinical efficacies and cognitive effects of MST (100 Hz applied over the vertex) and bifrontal ECT in patients with depressive episodes. We hypothesized that MST and ECT would result in similar improvements in depressive symptoms, yet MST would produce fewer cognitive side effects, such as memory loss.

## 2 MATERIALS AND METHODS

### 2.1 Study design and participants

The study was a rater‐blinded, non‐randomized comparative study. Eligible patients and their legal guardians decided which arm (MST or ECT) to enter for the clinical trial.

Participants were recruited from the outpatient and inpatient services of Beijing Anding Hospital, Capital Medical University (Beijing, China). The protocol was approved by the Ethics Committee of Beijing Anding Hospital, Capital Medical University. All participants or their direct family members were required to provide written informed consent before entering the study. The trial was registered in the Chinese Clinical Trial Register (ChiCTR‐ONN‐17010740) on February 27, 2017.

All participants ranged from 18 to 60 years of age and were required to meet the diagnostic criteria for depressive episodes, according to the Diagnostic and Statistical Manual of Mental Disorders‐IV‐TR (First et al., 2002). In addition, the score of the 17‐item Hamilton Rating Scale for Depression (HAMD‐17) had to be 17 or greater, and convulsive therapy was recommended by the clinicians (Hamilton, 1960). Diagnostic interviews were conducted by experienced psychiatrists who were trained before the assessment. Exclusion criteria for this study included comorbid psychiatric and medical conditions; substance abuse or dependence (except nicotine dependence); serious medical illnesses like cardiovascular, hepatic, renal, respiratory, hematological, endocrinological, head trauma, and seizure disorders; significant laboratory abnormalities; having received convulsive therapy or taking long‐acting anticonvulsant medications within 1 month of enrollment; female patients who were pregnant or planned to become pregnant; a history of anesthetic or muscle relaxant allergies, such as propofol, etomidate, or succinylcholine. In addition, patients with electromagnetic implants were excluded from the study. The concurrent use of SSRI medications was permitted; however, the participants were required to keep antidepressants on stable dosage throughout the study. The concurrent use of benzodiazepines was not allowed. Treatment discontinuation was decided by the patient's wish to withdraw from the study at any time or by psychiatrists based on medical complications or other conditions.

### 2.2 Patient assessments

Clinical symptoms were assessed using the HAMD‐17 and Hamilton Anxiety Rating Scale (HAMA) (Hamilton, 1959) by experienced psychiatrists who were trained before the assessment. The Repeatable Battery for the Assessment of Neuropsychological Status (RBANS) (Silverberg et al., 2007) was used to measure cognitive function. The RBANS generates five index scores for five neurocognitive domains, including immediate memory, visuospatial and constructional abilities, language, attention, and delayed memory. All assessors were blinded to the treatments of the participants. Participants were assessed three times, including before the treatment (baseline), after the third treatment session, and after the sixth treatment session. Ictal seizure durations were recorded by visual observation. Seizure duration, recovery time of breathing, consciousness, and orientation were recorded by the nurse or research assistant. The primary outcome measure was defined as the change in RBANS score between baseline and post‐treatment. The secondary outcome measures were the response rates and remission rates of MST and ECT.

### 2.3 MST treatment

Based on previous studies (Eric et al., 2015; Milev et al., 2016; de Sousa et al., 2015), the patients received a total of six treatment sessions in this study. The patients were treated on days 1, 2, 3, 5, 7, and 9. MST was administered using the MST stimulator and circular coil (Magstim Inc.). The coil diameter was 130 mm, and the international standard 10–20 EEG system was used to establish the position of the coil's center. The coil's center was placed over the vertex of the patient's head, in the middle site of P3 and P4. The stimulation frequency was 100 Hz, with 100% maximum stimulator output. The maximum stimulation duration was 10s (Eric et al., 2015; Milev et al., 2016; de Sousa et al., 2015). General anesthesia was induced with propofol (1.5 mg/kg) and muscle relaxation with succinylcholine (0.5 mg/kg).

### 2.4 ECT treatment

Based on previous studies (Boroojeny et al., 2012; Hirano et al., 2017; Kellner et al., 2015), participants received six treatment sessions of ECT with a bifrontal electrode in this study. The patients were treated on days 1, 2, 3, 5, 7, and 9. ECT was administered with a brief‐pulse constant current apparatus (Somatics Thymatron^®^) (Teraishi et al., 2012). The dosage was determined using the half‐age method in this study (Chanpattana et al., 2000). General anesthesia was induced with propofol (1.5 mg/kg), and succinylcholine (0.5 mg/kg) was used for muscle relaxation (Wang et al., 2012).

### 2.5 Statistical analysis

The statistical analysis was performed using the Statistical Package for the Social Sciences, version 22.0 (SPSS Inc.). Statistical significance was set at the level of 0.05, two‐tailed. Demographic, clinical, and cognitive data were shown as the mean ± *SD*. The demographic and clinical characteristics at baseline were compared between MST and ECT groups using the between‐group *t* tests. Frequency counts of response and remission status were compared using the *Chi‐square* test. Repeated measures analysis of variance (ANOVA) was applied to evaluate group and time‐dependent effects of MST and ECT on the scores of HAMD‐17 and RBANS. Pearson's correlation test was performed to examine the correlation between the changes in RBANS scores for the pre‐treatment and post‐treatment, seizure duration, breathing recovery time, consciousness recovery time, and orientation recover time.

## 3 RESULTS

### 3.1 Participants

A total of 52 patients were assessed for eligibility in this study. The patients were screened from Beijing Anding Hospital, Capital Medical University (Beijing, China) between March 2017 and September 2017. Six patients failed to meet the inclusion criteria (HAMD < 17) and were excluded from the study. In addition, one patient was excluded due to a suspected cerebral hemorrhage. Hence, 45 right‐handed patients with depressive episodes participated in the study, of which 18 received MST and 27 received ECT. All participants completed the entire study protocol and experienced treatment‐induced seizures successively during each session. The demographics and clinical characteristics of the participants, including age, gender, medication use, disease duration, and baseline clinical measures, were summarized in Table 1. There were no statistical differences in baseline characteristics between the two groups (*p* > .05).

**Table 1:** Demographics and clinical characteristics of enrolled patients (*n* = 45)

	MST group (*n* = 18)	ECT group (*n* = 27)	*t*/*χ* ^2^	*p*
Age, mean (*SD*), years	29.00 (8.32)	32.78 (8.84)	−1.437	.158
Sex				
Male, No. (%)	2 (11.11%)	5 (18.52%)	0.063	.801
Female, No. (%)	16 (88.89%)	22 (81.48%)		
Years of Education, mean (*SD*)	13.11 (3.10)	13.00 (3.41)	0.111	.912
Age at onset, mean (*SD*), years	24.72 (6.86)	24.96 (7.90)	−1.420	.163
Duration of illness, mean (*SD*), years	3.77 (3.77)	4.47 (5.42)	−0.474	.683
Frequency of depressive episodes, mean (*SD*), times	2.22 (1.73)	2.63 (2.04)	−0.695	.491
Frequency of hospitalizations, mean (*SD*), times	1.39 (0.61)	1.63 (1.18)	−0.795	.431
Family history of mood disorders, No. (%)	9 (50%)	12 (44.44%)	0.134	.714
Lifetime suicidal ideation/attempts, No. (%)	15 (83.33%)	21 (77.78%)	0.006	.939
Complications, No. (%)	5 (27.78%)	8 (29.63%)	0.018	.893
Medications				
Antidepressants, No. (%)	13 (68.42%)	19 (70.37%)	0.018	.893
Antipsychotics, No. (%)	10 (55.56%)	13 (48.15%)	0.237	.626
Mood stabilizers, No. (%)	7 (38.89%)	10 (37.04%)	0.016	.900
Baseline clinical symptom and cognitive function				
HAMD‐17 total scores, mean (*SD*)	27.39 (7.31)	26.81 (5.91)	0.290	.773
HAMA‐14 total scores, mean (*SD*)	29.56 (10.42)	28.67 (8.55)	0.313	.756
RBANS, mean (*SD*)				
Immediate memory	80.28 (18.17)	76.26 (17.13)	0.753	.456
Visuospatial	89.67 (14.54)	96.19 (17.88)	−1.287	.205
Language	95.94 (14.36)	92.78 (15.83)	0.682	.499
Attention	90.72 (15.52)	90.85 (17.40)	−0.026	.980
Delayed memory	86.44 (22.66)	84.37 (20.38)	0.320	.751
Total score	85.67 (16.60)	85.11 (16.60)	0.110	.913

Abbreviations: HAMA, Hamilton Anxiety Scale for 14‐items; HAMD‐17, The Hamilton Depression Rating Scale for 17‐items; RBANS, Repeated Battery for the Assessment of Neuropsychological Status.

### 3.2 Clinical outcomes

**FIGURE 1:**
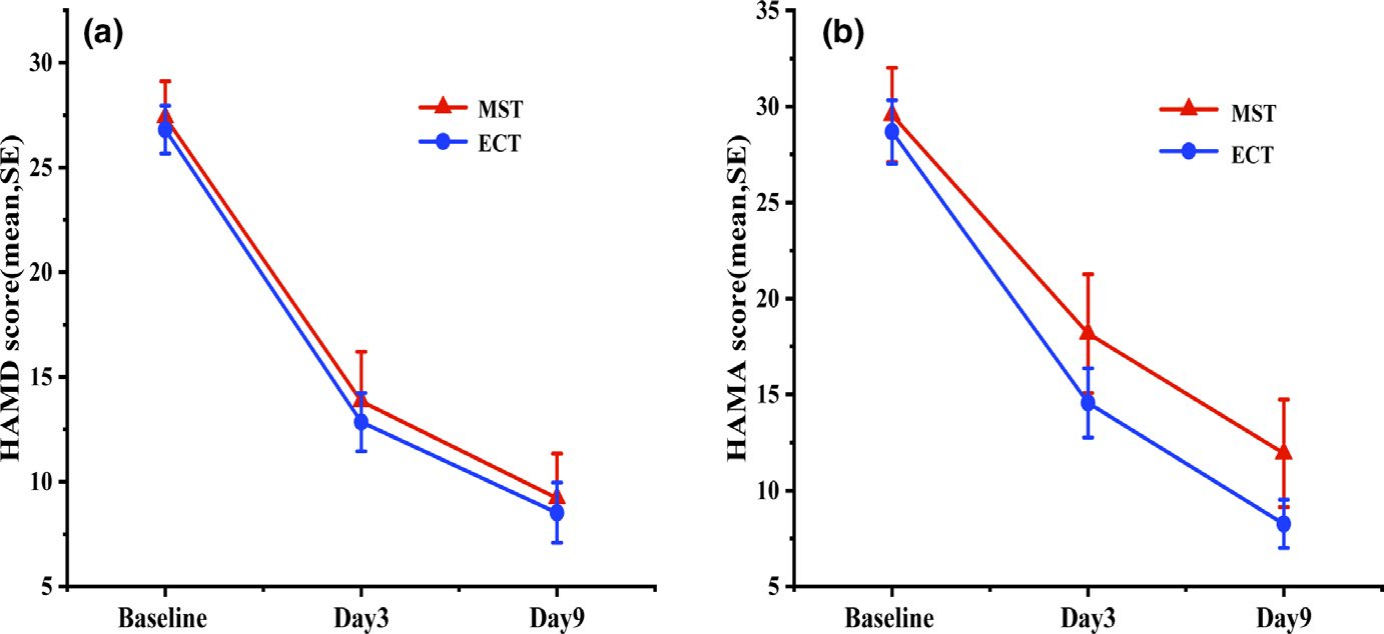
The trend of HAMD and HAMA at baseline, 3 treatment, 6 treatment

In both groups, significant reductions were detected in the severity of depressive symptoms (HAMD‐17: *F* = 152.933, *p* < .001) and anxiety symptoms (HAMA: *F* = 83.397, *p* < .001) after treatment compared with baseline. There were no significant differences between two groups in terms of depression symptoms (HAMD‐17: *F* = 0.018, *p* = .982), anxiety symptoms (HAMA: *F* = 0.564, *p* = .571), comparing the change of pre‐ and post‐treatment between the two treatment groups (Table 2). The reductions of HAMD‐17 and HAMA from baseline were significant after three treatment sessions in both groups, but no significant differences were detected between the two treatment groups (Figure 1).

**Table 2:** Indexes of primary and secondary outcomes before and on days 3 and 9 of treatment

	Baseline		Day 3		Day 9		Interaction size	
	MST group (*n* = 18)	ECT group (*n* = 27)	MST group (*n* = 18)	ECT group (*n* = 27)	MST group (*n* = 18)	ECT group (*n* = 27)	*F*	*p* (group × time)
HAMD‐17^a^, ^d^	27.39 (7.31)	26.81 (5.91)	13.83 (10.07)	12.85 (7.24)	9.22 (9.01)	8.52 (7.48)	0.018	.982
HAMA‐14^a^, ^d^	29.56 (10.42)	28.67 (8.55)	18.17 (13.09)	14.56 (9.35)	11.94 (11.88)	8.26 (6.54)	0.564	.571
RBANS								
Immediate memory^a^, ^b^, ^c^, ^d^	80.28 (18.17)	76.26 (17.13)	104.22 (26.00)	87.11 (20.41)	112.33 (25.17)	88.26 (22.51)	11.115	˂.001
Visuospatial/constructional^a^, ^c^	89.67 (14.54)	96.19 (17.88)	90.78 (20.06)	97.52 (15.45)	101.22 (11.94)	100.15 (12.86)	1.465	.237
Language	95.94 (14.36)	92.78 (15.83)	97.28 (13.02)	92.96 (14.26)	98.67 (15.66)	88.67 (13.36)	1.464	.238
Attention^a^, ^c^	90.72 (15.52)	90.85 (17.40)	96.50 (16.05)	92.48 (18.82)	102.94 (15.33)	91.26 (21.40)	4.240	.026
Delayed memory^a^, ^b^, ^c^, ^d^	86.44 (22.66)	84.37 (20.38)	97.67 (14.17)	85.70 (20.96)	108.67 (14.58)	86.04 (21.03)	7.951	.002
Total score^a^, ^d^	85.67 (16.60)	85.11 (16.60)	96.61 (19.95)	88.78 (18.97)	106.72 (19.02)	88.19 (18.37)	11.052	˂.001

Notes

Means and standard deviations are presented.

Abbreviations: HAMA, Hamilton Anxiety Scale for 14‐items; HAMD‐17, The Hamilton Rating Scale for Depression; RBANS, Repeated Battery for the Assessment of Neuropsychological Status.

a Time effect was significant. ^a^

b Group effect was significant. ^b^

c *p* ˂ .05. *

d *p* ˂ .01. **

**FIGURE 2:**
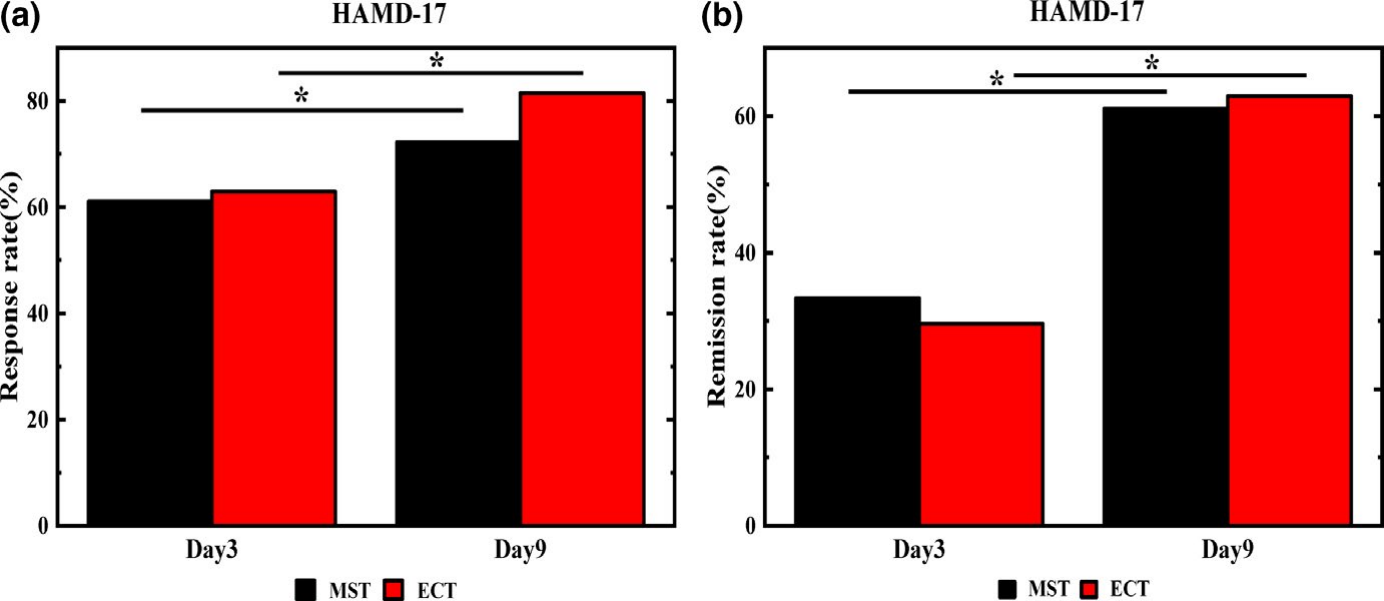
HAMD response rates and remission rates of patients in the MST and ECT groups

Treatment response was defined as >50% improvement in HAMD, as compared with the baseline values and a remission was defined when HAMD‐17 ≤ 7. After three treatment sessions, there were no significant differences between MST and ECT groups in response rates (61.11% vs. 62.96%, respectively, *p* > .05) or remission rates (29.63% vs. 33.33%, respectively, *p* > .05). After six treatment sessions, there were no significant differences between the MST and ECT groups in response rates (72.22% vs. 81.48%, respectively, *p* > .05) and remission rates (61.11% vs. 62.96%, respectively, *p* > .05), as shown in Figure 2.

### 3.3 Duration of seizures

**FIGURE 3:**
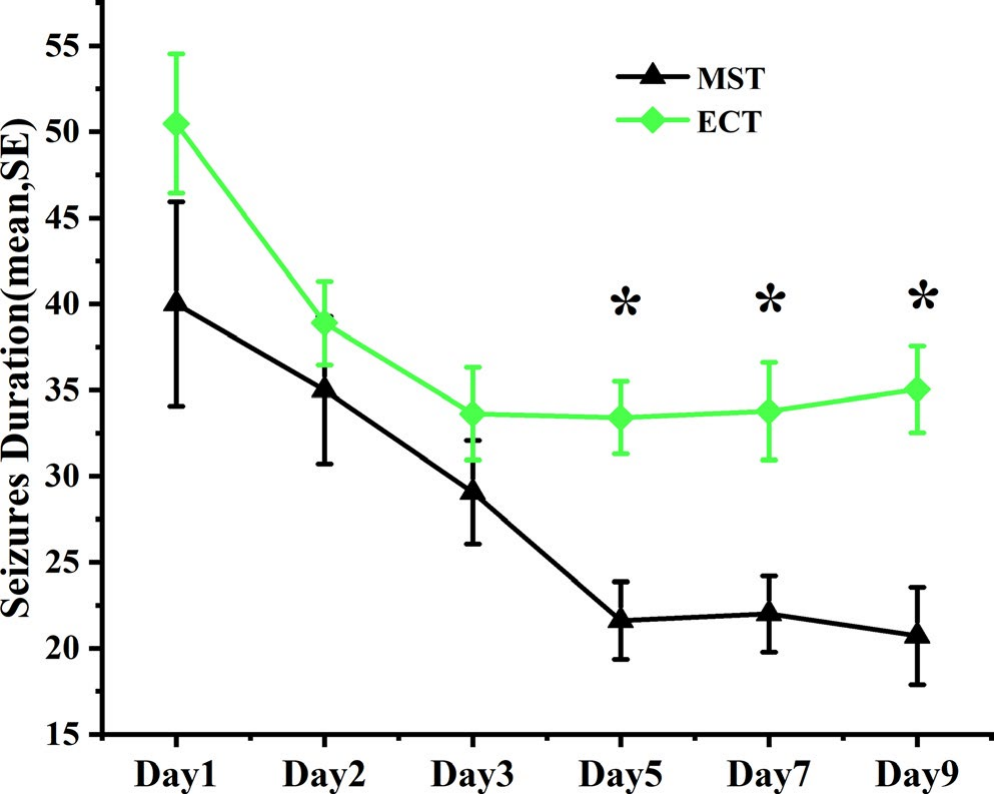
Comparison of seizure durations between MST and ECT groups

The average seizure duration for treatments 4, 5, and 6 was longer in ECT group than MST group (*F* = 7.651, *p* = .008). There were no significant differences in seizure duration for treatments 1, 2, and 3 between the two treatment groups (Figure 3).

### 3.4 Recovery times for breathing, consciousness, and orientation

**FIGURE 4:**
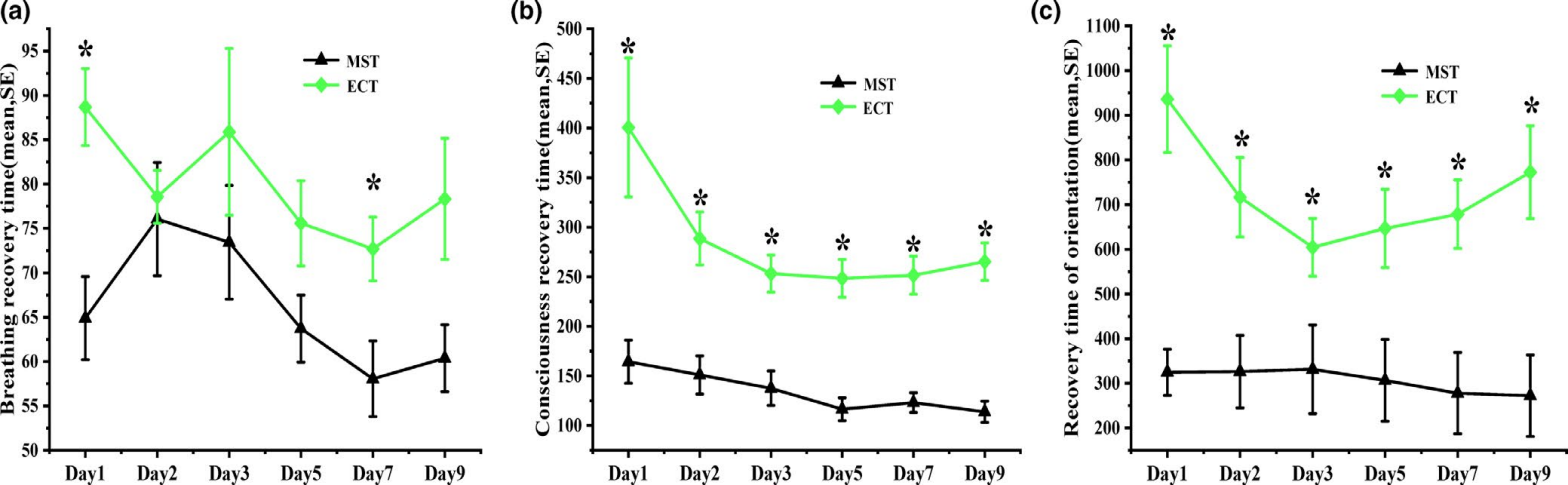
Recovery times for breathing, consciousness, and orientation in MST and ECT groups

Time to orientation is the time from the end of the seizure to patients regaining their sense of orientation. There were significant differences in the recovery time of breathing (*F* = 6.696, *p* = .013), consciousness (*F* = 22.48, *p* < .001), and orientation (*F* = 14.688, *p* < .001) between the two groups (Figure 4). The recovery times of breathing, consciousness, and orientation were significantly shorter in MST group, as compared with ECT group.

### 3.5 Cognition: RBANS score

After undergoing treatment on days 3 and 9, the composite scores for RBANS in MST group were significantly improved in comparison to the baseline values, and the improvement was significantly better than ECT group as the group time interaction was significant (*p* < .01). However, there was no significant change in RBANS scores in ECT group after days 3 and 9 treatment (*p* > .0.5).

In addition to improvement in the composite RBANS scores, the three dimensions of immediate memory, delayed memory, and attention were significantly improved in MST group after days 3 and 9 treatments, and these values were significantly better than those of ECT group (group time interaction was significant, *p* < .01). There was no significant change in the cognitive function of patients in ECT group (*p* > .05). In MST group, there were no significant differences in the two dimensions of speech and visual spatial memory between the days 3 and 9 treatments (*p* > .05), and there were no significant improvements in the scores of the two groups after treatment (Table 2).

### 3.6 Correlation RBANS scores for seizure duration, recovery times of breathing, consciousness, and orientation

Pearson correlation analysis revealed no significant associations between changes in total RBANS scores for seizure duration and recovery times of breathing, consciousness, and orientation between baseline and after treatment. Positive correlations were found between the recovery time for orientation and seizure duration (*r* = .561, *p* < .001), recovery time for breathing (*r* = .361, *p* = .015), and recovery time for consciousness (*r* = .726, *p* < .001). There was no significant correlation between the recovery time of orientation and seizure duration, the recovery time of breath, the recovery time of consciousness. Using seizure duration and the recovery time for breathing and consciousness as control variables, the results showed that the recovery time for orientation and changes in total RBANS scores were negatively correlated (*r* = −.309, *p* = .047). Using RBANS total score as dependent variable, seizure duration, and the recovery time for breathing, consciousness, and orientation as independent variables, the regression equation was Ŷ = 18.144–0.014x. Hence, the differences between RBANS total scores decreased by 0.014 on average for each unit of increase in the recovery time of orientation. The recovery time for orientation could explain the variance of RBANS total scores.

## 4 DISCUSSION

Magnetic seizure therapy is a new type of convulsive therapy that induces seizures similar to ECT, but with greater control over the induction and spread of the seizures (Fitzgerald et al., 2018; Kayser et al., 2020). In this study, we found no significant differences in clinical outcomes between MST and ECT, and response rates and remission rates were numerically better for patients receiving ECT. The response rates were 72.2% and 81.5% for MST and ECT, respectively. The remission rates were 61.1% and 63.0%, respectively. The findings are consistent with previous reports in the literature, showing that the response rates of MST range from 50% to 75% (Backhouse et al., 2018; Eric et al., 2015; Polster et al., 2015; Radman & Lisanby, 2017).

Our findings are consistent with previous studies concerning the impact of MST on cognition (Cycowicz et al., 2009; Mcclintock et al., 2011). We observed no cognitive impairments with ECT, and we found cognitive improvements with MST. First, the effect on memory and attention was significantly different between MST and ECT treatment groups (Cycowicz et al., 2009, 2017; Lisanby et al., 2003; Mcclintock et al., 2011). MST has been shown to improve both immediate and delayed memory, along with attention (Cycowicz et al., 2009, 2017; Lisanby et al., 2003; Mcclintock et al., 2011). Since there was no significant difference in antidepressant efficacy between MST and ECT, the improvements in cognitive function were less likely due to the improvement of depressive symptoms. The differences in the effect of cognitive function between MST and ECT may be related to their different mechanisms on brain function (Lee et al., 2017).

Magnetic seizure therapy affects the brain function of the stimulation site directly, while other brain areas are impacted through anatomical and functional connectivity (Atluri et al., 2018; Deng et al., 2013). ECT produces its therapeutic effect through two electrodes acting on the brain, so it impacts more extensive areas of the brain, including deep brain areas like the hippocampus, which is why ECT can lead to memory loss. MST and ECT have similar effects on α, β, and γ waves, yet the effect on θ waves is more significant on ECT than MST. Hence, θ waves may be related to cognitive function (Eitan & Lerer, 2006; McClintock et al., 2013; Peterchev et al., 2015).

Finally, a few limitations of this study should be acknowledged, including the non‐randomized design, the relatively small sample, and the relatively limited numbers of treatments. Additionally, the electrode placement and dose of electricity in the ECT group might have contributed to more severe side effects in the group.

## CONFLICTS OF INTEREST

The authors declare that they have no conflict of interest.

## AUTHOR CONTRIBUTIONS

JZ, YR, WJ, YT, and XM designed the study and contributed to data acquisition, analysis, and interpretation. JL and FY contributed to clinical evaluation. All authors contributed to the article and approved the submitted version.

## ETHICS APPROVAL AND CONSENT TO PARTICIPATE

The protocol was approved by the Ethics Committee of Anding Hospital, Capital Medical University. All participants or their direct family were required to provide written informed consent before entering the study.

## CLINICAL TRIAL REGISTRATION

The trial was placed in the Chinese Clinical Trial Register (ChiCTR‐ONN‐17010740) on February 27, 2017.

## Data Availability

The datasets generated and analyzed during the present study are available from the corresponding author on reasonable request.
